# Spatially balanced provision of health equipment: a cross-sectional study oriented to the identification of challenges to access promotion

**DOI:** 10.1186/s12939-017-0704-x

**Published:** 2017-12-04

**Authors:** Pedro Vasconcelos Amaral, Thiago Augusto Hernandes Rocha, Allan Claudius Queiroz Barbosa, Adriana Lein, João Ricardo Nickenig Vissoci

**Affiliations:** 1 Centre for development and regional planning, Federal University of Minas Gerais, Belo Horizonte, Minas Gerais Brazil; 2 Center of post-graduate and Research in Administration Belo Horizonte, Federal University of Minas Gerais, Faculty of Economics Sciences, Belo Horizonte, Minas Gerais Brazil; 3 Faculty of Economics Sciences, Department of Business Administration, Federal University of Minas Gerais, Belo Horizonte, Minas Gerais Brazil; 4 Division of Emergency Medicine, Duke University Health System, Duke Global Health Institute,, Duke University, North Carolina, Durham, USA

**Keywords:** Spatial analysis, Hospitals, Health care evaluation mechanisms

## Abstract

**Background:**

Access to health services is in part defined by the spatial distribution of healthcare equipment. To ensure equity in the provision of health services, it is important to examine availability across different health care providers taking into account population demand. Given the importance of the equitable provision of health equipment, we evaluate its spatial distribution in Brazil.

**Methods:**

This study is classified as cross-sectional with an ecological design. We evaluate Brazilian data on distance to available health equipment considering: dialysis machines (385), magnetic resonance imaging (MRI) (257), hospital beds (3675) and bone densitometers (429). We define two distance thresholds (50 km and 200 km) from a municipality to the center of services provision. The balance between infrastructure capacity and potential demand was evaluated to identify a lack or surplus of health services.

**Results:**

The distribution of dialysis equipment and bone densitometers is not balanced across Brazilian states, and unmet demand is high. With respect to MRIs, the large capacity of this equipment results in a large excess of supply. However, this characteristic alone cannot account for excesses of supply of over 700%, as is the case of the Federal District when the range is limited to 50 km. At the same time, four states in the Northeastern region of Brazil show a net excess of demand. Some regions do not meet the standard amount of supply defined by Brazilian Ministry of Health. The quantity and distribution of hospital beds are not sufficient to provide full coverage to the population.

**Conclusion:**

Our main focus was to evaluate the network of the provision of health equipment in Brazil, considering both private and public sectors conjointly. We take into account two main aspects of a spatially balanced health system: the regional availability of health equipment and the geographic distance between its demand and supply at the municipality level. Some regions do not meet the minimum requirement defined by the Brazilian Ministry of Health regarding the supply of health services.

## Background

The geographic accessibility to health services is increasingly regarded as an important field of study for policy planners and researchers [1, 2]. Geospatial unbalances in the provision of health services are the result of inadequate spatial distribution of people and health equipment [3]. Usually, health services are provided in a limited number of locations, yet they serve populations unevenly distributed throughout a specific region [4]. Inequalities regarding the geographical accessibility are inevitable, but their magnitude could be addressed through effective analysis of health equipment distribution [3, 5].

Improvements in the spatial arrangement of a health care delivery system have the potential to decrease such inequalities [3]. The main approach, to produce evidence capable to support policies dedicated to decreasing inequalities in geographic accessibility is based on the examination of different scenarios of distances between people and health care facilities [3]. Accessibility is a measure of the friction of distance among locations, whereas availability generally measures the number of services in comparison to the number of potential users of the service [3]. Understanding how the distribution of health equipment is related to limited spatial access creates conditions for health planners to simulate the impact of changes in the healthcare network [6]. Hence, the better understanding of the spatial accessibility, the better will be the capacity to promote improvements capable to decrease this lack of access.

Brazil’s Unified Health System (SUS) establishes that access to health services is guaranteed to all citizens, with the right to full coverage for medical needs and equitable treatment. One of the requirements of horizontal equity is that people have balanced access to health services in order to receive diagnosis or treatment. In this context, balanced access to health services implies that equal access is provided for persons with equal needs. Therefore, accessibility is more than the existence or availability of resources at any given time [5]. It is related to the capacity of individuals to effectively utilize the services provided. Several factors can restrict or impede access to health services, such as insurance coverage and level, language and cultural differences, the geographic distribution of health services, education level, which includes health literacy and socioeconomic status, and transportation costs, among others. These factors can be broadly classified in two main categories of socio-organization and geographic accessibility [7].

Under the umbrella of geographic accessibility, the spatial distribution of health services assumes an important role, together with the transportation system. This is because service provision and the patient’s ability to reach or be reached by health services must be concurrent. Considering geographic perspectives, accessible health services are those which “enable people to travel to them at reasonable cost, in reasonable time and with reasonable ease" [8]. The traditional view in economic theory is that there is a trade-off between equality and efficiency [9]. The debate on the equality versus efficiency trade-off is very relevant to the discussion of locational strategies in allocating public goods [9]. In the specific case of the allocation of health facilities, the trade-off translates in balancing efficiency of public spending on health and equality of access. From a social justice point of view, access to treatment should be available within a reasonable distance for all individuals, regardless of population’s characteristics and geographic location.

Few studies explicitly address the role of geographic distance in the provision of health services in Brazil, and existing studies are confined to analyses of single states or regions. These studies demonstrate how the probability of being hospitalized is inversely correlated with the distance between the city where a hospital is located and the patient’s hometown [10]. This is aggravated by the concentration of hospitals in more developed urban areas and access gaps in rural areas and the Amazon region, leading to the conclusion that distribution of emergency services in Brazil is not facilitating access to the population due to geographical barriers associated with great distances [11]. The literature about access to specific equipment in even scarcer. For mammography equipment, evidence shows that overall provision in the country should be sufficient to cover all population. However, given spatial concentration, several regions of the country lie outside a provision area [12].

To help filling this gap in the literature, this study aims to evaluate the provision of health equipment in Brazil based on the two main aspects of a spatially balanced health system: the regional availability of health equipment and its potential accessibility measured through the geographic distance between its demand and supply at a municipality level.

## Methods

The present study is classified as cross-sectional with an ecological design. Due to its multi-scale nature, the health services considered in this work range from medium complexity (e.g. hospital beds or bone densitometers), to high complexity (e.g. dialysis machines and magnetic resonance imaging (MRI) equipment). The selection of these types of health equipment is due to their presence on a policy bill formulated by Brazilian Ministry of Health. This bill establishes parameters for the provision of several types of health equipment, professionals, and procedures according to population size. These parameters provide the basis for the calculation of a standard provision amount for each category of equipment.

Table 1 presents descriptive statistics regarding the number of items of health equipment available in Brazil. All data on health equipment correspond to December 2010 and are provided by the Ministry of Health, through its Information Technology Department – DATASUS [4]. As Table 1 shows, of the 5565 Brazilian municipalities, 3675 have hospital beds, 429 municipalities have bone densitometers, and dialysis equipment and MRIs are concentrated in 385 and 257 municipalities, respectively.

**Table 1:** Health equipment in Brazil

Variable	Number of municipalities^1^	Mean	Std. dev.	Min.	Max.	Standard supply of the equipment [15]
Bone densitometer	429 (7,71)	3,8	12,27	1	202	1 for each 14,000 inhabitants
Dialysis equipment	385 (6,92)	47,14	114,1	1	1685	1 for each 1500 inhabitants
Hospital beds	3675 (66,05)	126,03	751,95	1	29,562	2.5 to 3 beds for each 1000 inhabitants^2^
Magnetic resonance imaging	257 (4,62)	4,38	11,46	1	130	1 for each 500,000 inhabitants

^1^Percentage of municipalities in parenthesis

^2^In this paper, we assume 2.75 hospital beds for each 1000 population

### Study area

The standard amount of provision for health equipment as defined by the Brazilian Ministry of Health is utilized in this study as a measure of the capacity of the equipment – Table 1. All 5565 Brazilian municipalities were considered in our study. These municipalities totaled 190,755,799 people [5]. The data on population is provided by the Demographic Census of 2010, published by IBGE [5]. All data used were public available.

### Data analysis: Categorization of surplus or demand for municipalities considering the provision of services for each equipment

The justification for categories of equipment included in this paper is based on data availability, information on the standard amount of supply, and a representativeness of equipment of both medium and high complexities. The analysis of the spatial distribution of health equipment yields information for both private and public policymakers, and shows a status of the provision of these types of equipment within the Brazilian health system.

Given the amount of equipment available at any locality and its capacity, we are able to estimate its potential supply by defining how many patients could potentially be served by that locality. Considering population size (p) as a measure of demand and the standard amount of supply (s) as a measure of the capacity of the health equipment, both demand and supply can be estimated. These formulas allow for the estimation of the excess of demand or supply (E) of each municipality i and health equipment k:

$$ {E}_{\mathrm{ki}}={h}_{\mathrm{ki}}{s_k}^{-1}-{p}_i $$

in which (*h*) represents the amount of items of equipment, (*s)* is the standard amount of supply, as defined by the Ministry of Health, and p the population size. For example, a municipality counting with 100,000 people (p), ten bone densitometer(h) with the capacity to cover 140,000 (standard amount 1 for each 14,000 inhabitants), will be categorized as in a surplus situation, once the total population covered is sufficient to cover the entire population of the municipality, with a surplus of 40,000 inhabitants. This calculation was repeated considering each equipment and for each municipality. Despite the importance of the access to a specific type of health equipment, we acknowledge that there are limits to the distance that the patient is expected to travel in order to reach a healthcare provider. We consider four different levels for the maximum range to a locality with surplus of health services (Eki > 0): 50 km, 100 km, 150 km, and 200 km. These four ad hoc distance thresholds were chosen to illustrate how gradually the coverage area evolves with wider distance ranges and to identify the gaps in the provision of the health services that would persist even when such an extreme maximum range as 200 km is considered. Our objective is to approximate the average distance to the nearest neighboring municipality. Furthermore, we are interested in examining the maximum one-way distance that an individual can feasibly travel in one day to reach a location with a health center and return home without spending the night^1^. The neighboring region of a healthcare provider is constrained by these distances. The range is a measure of how far the surplus of the reference locality can be used to assist with the deficit from the nearest neighbors, taking into account the maximum distance restriction. If a locality with surplus is surrounded by neighbors that present deficits, the range of municipality with surplus can be calculated as the distance to the nearest neighbor with a deficit added to the deficits of all close neighbors to compensate for reference locality’s surplus.

The distance between municipalities is calculated according to the great circle distance between geographic coordinates of the primary district of each municipality. This approach is commonly used to assess distance patterns between the population and health care facilities [13]. The great circle distance formula is used to identify the shortest path between two points on the surface of a sphere [14]. Given the small spatial scale of our units of analysis, we assume no transportation costs within the locality of reference.

**Fig. 1:**
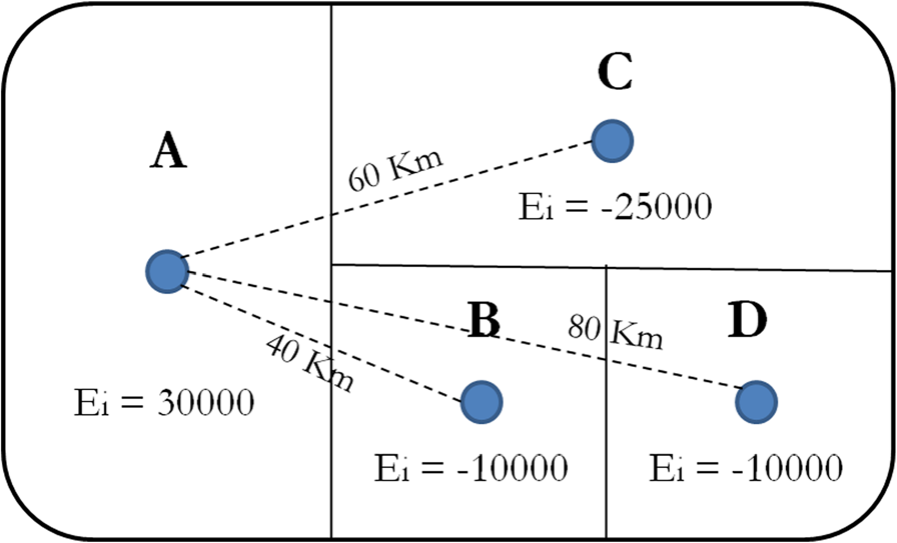
The range of a fictional health care network

In practice, we assume that the population and health equipment in a given locality are located at the same point in space, this point being the geographic coordinates of the primary district of the municipality. The coordinates of the primary district are chosen rather than centroids because they reflect the actual urban center within the municipality. Given that population and equipment within a municipality are considered to be at the same point in space, the distance between them is zero. Therefore, the distance between two municipalities is the great circle distance from the coordinates of the primary district of the municipality of origin to its border, added to the distance from the border to the coordinates of the primary district of the municipality of destination. An example is given in Fig. 1.

The center A is the only locality with an excess of a supply of health equipment. The surplus in A is EA = 30,000, while the localities B, C and D present deficits of EB = −10,000, EC = −25,000, and ED = −10,000. The distances between each of the localities are also shown in Fig. 1. Given that B is the nearest neighbor of A and given the amount of excess of supply and demand from each, we can expect the population of B to be served by the provision in A. If all the inhabitants of B receive health care in A, the surplus in A would be reduced to EA + B = 20,000. The next nearest neighbor of A is C. Since the distance between A and C is 60 km, if we use a maximum range of 50Km, the excess of supply at A would be beyond reach of the population from C, which would remain unassisted and A would end up with an excess supply of 20,000. However, if the maximum range is greater than 60 km, 100 km for example, the surplus in A can be used to provide healthcare to some of the inhabitants of C, although not to all. C would therefore be under the range of provision of A, but only partially served. If all the inhabitants of C receive health care in A, the surplus in A would disappear in EA + B + C = −5000. Since A would no longer have a surplus, none of its health care equipment would be available to assist the inhabitants of the locality D, which would remain not covered.

The objective of this methodology is highlight municipalities with a lack of capacity regarding the evaluated equipment. Additionally, investigate at which distance is the nearest municipality with surplus, that could act potentially act as a provision center, This way, the clusters of municipalities with excess demand is highlighted and the results identify the regions that either need another provision center to meet their demand, or an increase in the capacity of a provision center within the maximum range.

The demand versus surplus analysis was performed initially at the state level and after at the municipality level. The percentage of population covered in municipality level analysis was highlighted based on the results obtained through the E_ki_ variable detailed earlier. Four categories were defined to define the municipality situation: 0% - corresponding to no equipment available, 0% < X < 100% for the situations where the population is partially covered by the equipment available, 100% referring to an installed capacity to cover the entire population of the city and >100% for provision centers, capable to cover its entire population and, potentially, offer services to near municipalities.

## Results

### State level analysis

**Fig. 2:**
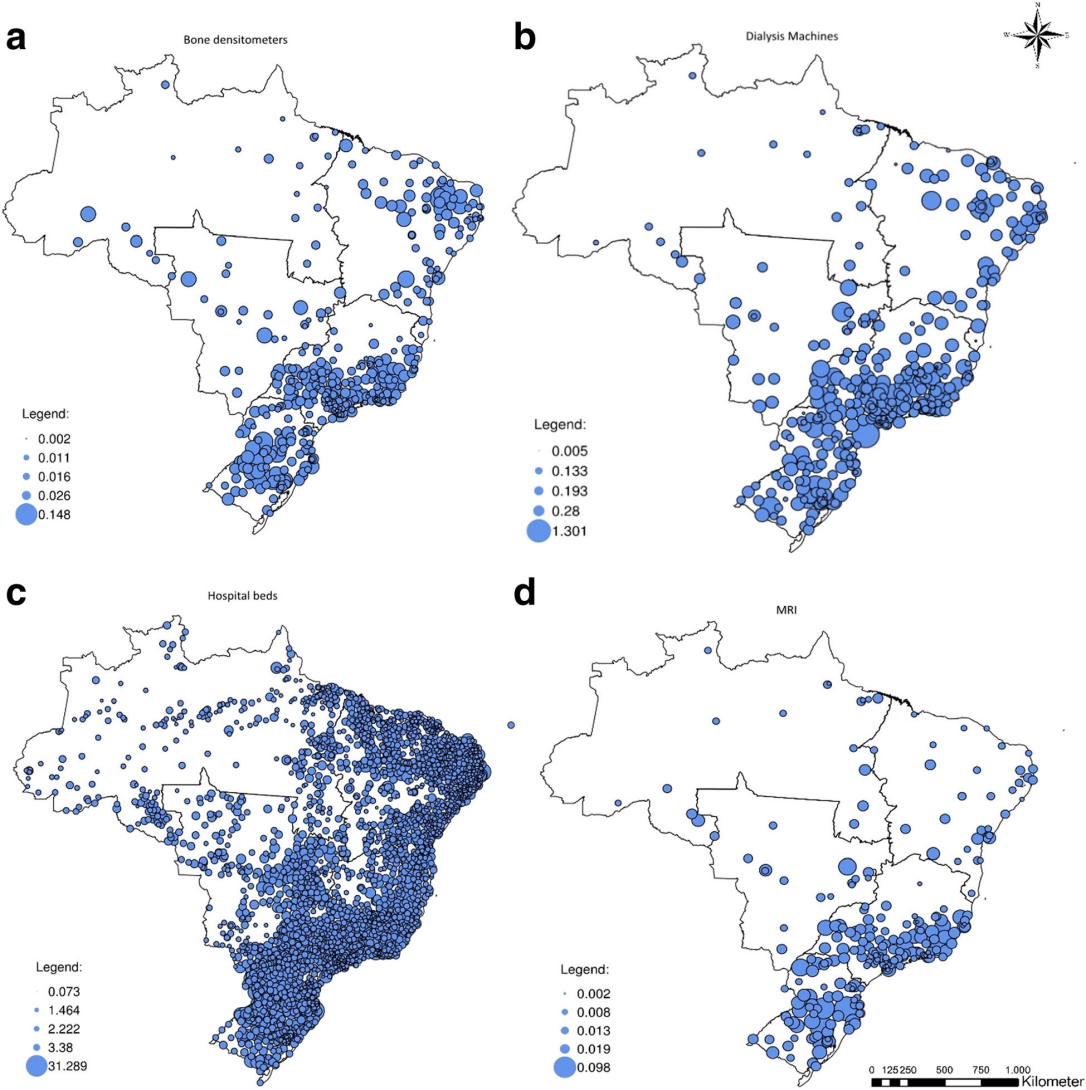
Spatial distribution of health equipment, normalized by population/10^3^

Figure 2 shows the spatial distribution of item of equipment per capita for bone densitometers (a), dialysis machines (b), hospital beds (c), and MRIs (d). In each map in Fig. 2, the smallest value in the legend represents the municipality with the smallest amount of equipment, considering the municipalities with at least one equipment available. In turn, the largest circle indicates the greatest amount available in a single municipality. Central-West, Southern, and South-Eastern regions of Brazil possess the largest share of equipment. Bone densitometers, dialysis machines and, markedly, MRIs are concentrated in these aforementioned regions. On the other hand, hospital beds are more distributed considering the whole country. Despite the more dispersed distribution of hospital beds, it was possible to observe disparities considering the volume of beds available between the country regions.

**Fig. 3:**
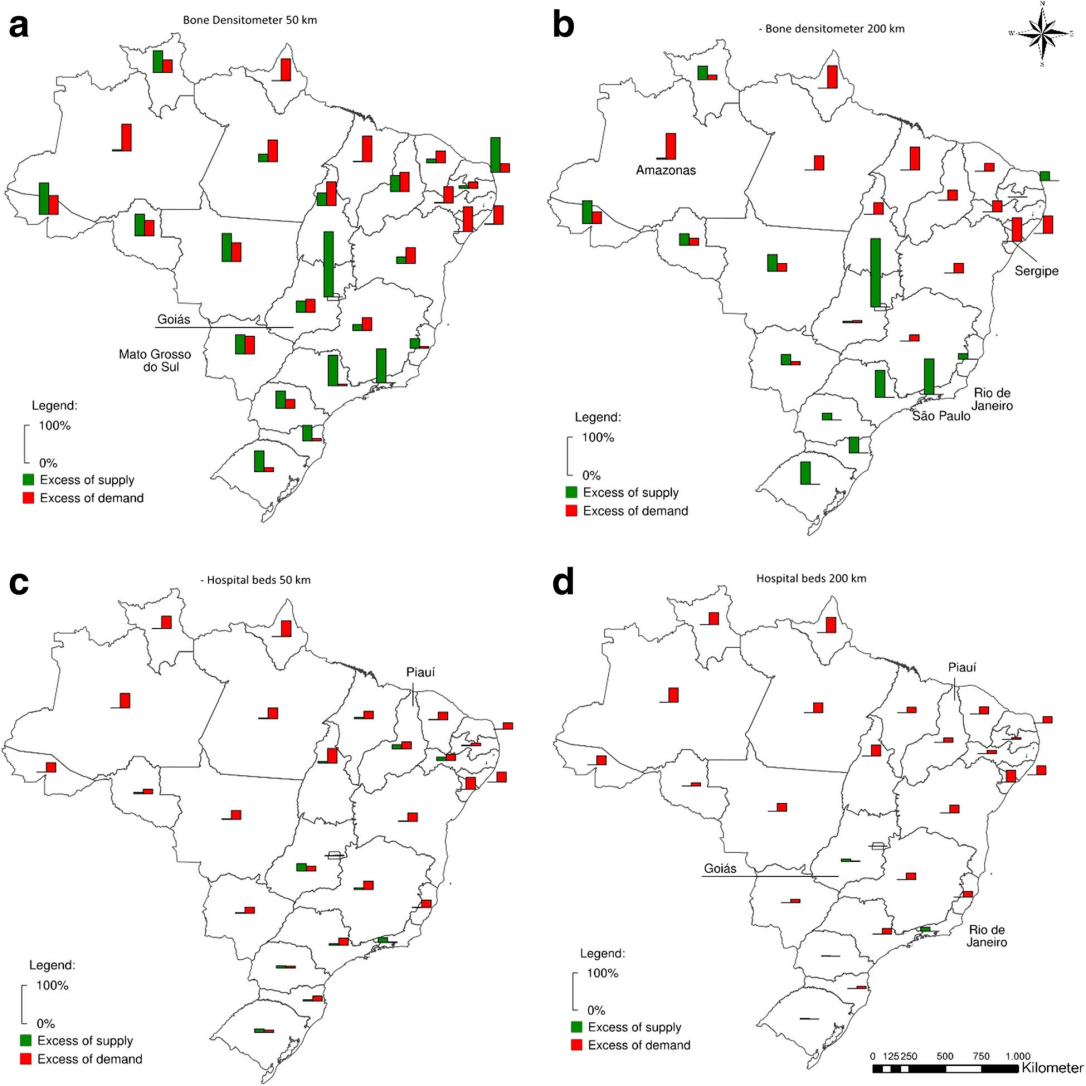
Excess of supply and demand considering maximum ranges of 50 km and 200 km in terms of % of state population: bone densitometers and hospital beds

**Fig. 4:**
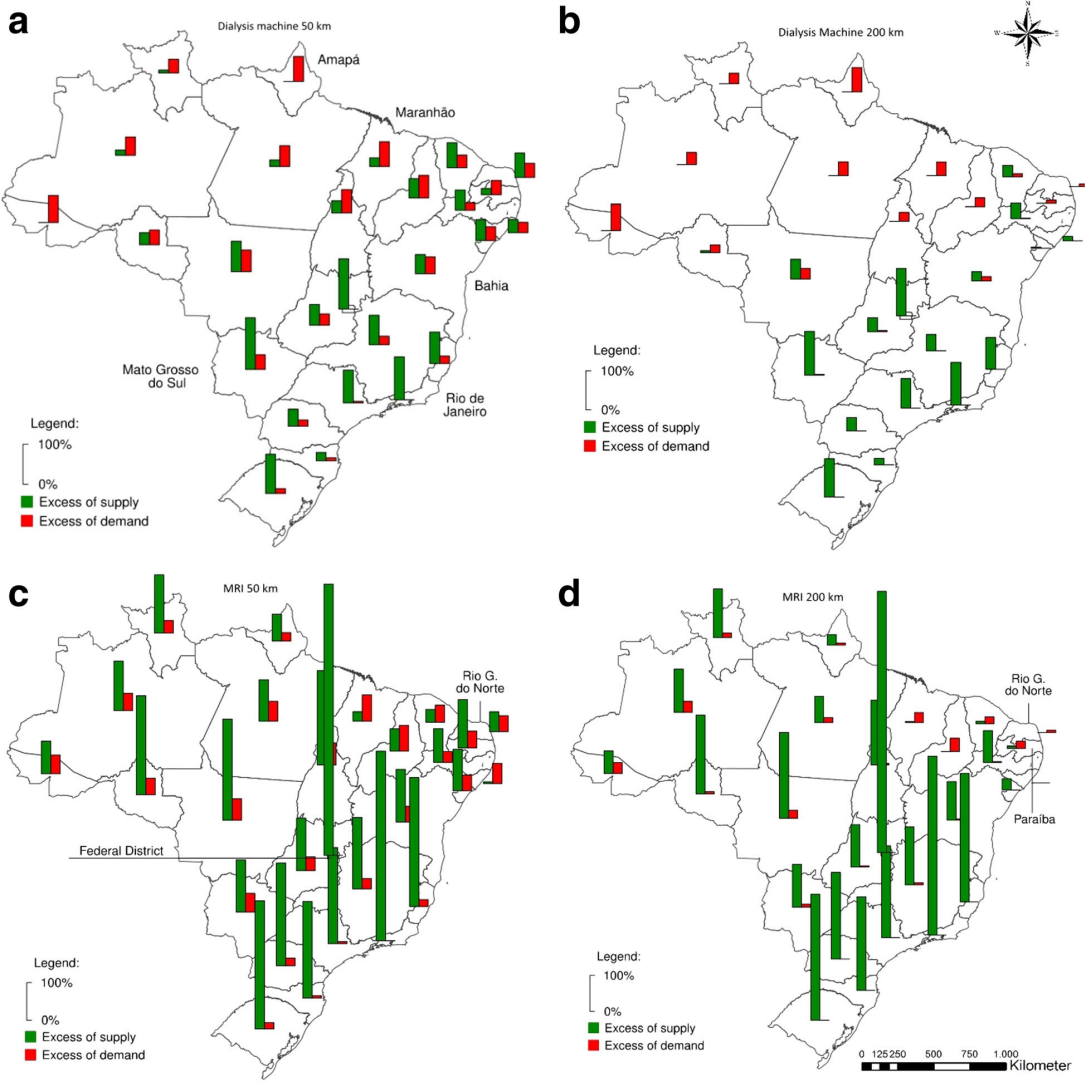
Excess of supply and demand considering maximum ranges of 50 km and 200 km in terms of % of state population: dialysis machines and MRI

Figures 3 and 4 show the distribution of an excess of supply and an excess of demand between the Brazilian states, for bone densitometer (3a-3b), hospital beds (3c - 3d), dialysis machines (4a-4b), and MRIs (4c-4d) under maximum ranges of 50 km and 200 km. When the maximum range of health care providers is constrained, the excess of demand for bone densitometers is concentrated in the Northern and Northeastern regions. Given the tendency for the equipment to be located in state capitals or major urban centers, when the limit is set to 50 km several states experience a concurrent excess of demand and supply. Few states, like São Paulo and Minas Gerais, remain with a concurrent excess of demand and supply when the range is limited to 200 km.

Regarding hospital beds, the problem is more one of lack of supply than distribution (Fig. 3). Even when a restrictive limit of 50 km is imposed, only 5 out of 27 states (including the Federal District) show an excess supply of over 5%. One of the poorest states in Brazil, Piauí, is among these five. Its capital alone accounts for 37.3% of all hospital beds in the state, while holding 26.4% of the population. Consequently, 18.4% of its population lives in areas farther than 50 km from any center of provision of hospital beds, whereas there is an excess supply of 11.5% in the state. This situation does not persist when the range’s limit is increased to the extreme of 200 km. All the excess supply from Piauí is used by both its own population and its neighbors. In this scenario, all the states in the North and Northeastern regions show some excess of demand, averaging 21.5%, and zero instances of excess supply. Only Rio de Janeiro and Goiás present an excess of supply of hospital beds greater than 2% when 200 km is considered as the maximum range.

The spatial structure of the provision of dialysis machines is similar to that of bone densitometers with a 50 km maximum range. In this case; however, most of the states in Northeastern Brazil present with a net surplus of equipment. If the range threshold is expanded to 200 km, most of the concurrent excess of demand and supply within states disappeared. In this scenario, the over concentration of equipment in South and Southeastern Brazil is evident. All states in the North present net excess demand, along with four in the Northeast. Even when such a high range as 200 km is considered, three states remain with more than enough excess supply to meet at least double their population’s needs.

With respect to MRIs, the large capacity of the equipment transfers to excess supply - its standard amount of supply is of 1 for every half million people [15]. However, this characteristic alone cannot account for excesses supply of over 700%, as is the case of the Federal District when the range is limited to 50 km. At the same time, four states in the Northeastern region of Brazil experience a net excess demand. When the maximum range is 200 km, this number is increased to five states, since part of the surplus of Rio Grande do Norte and Paraíba is used to assist the population of its neighbors.

### Municipality level analysis

**Fig. 5:**
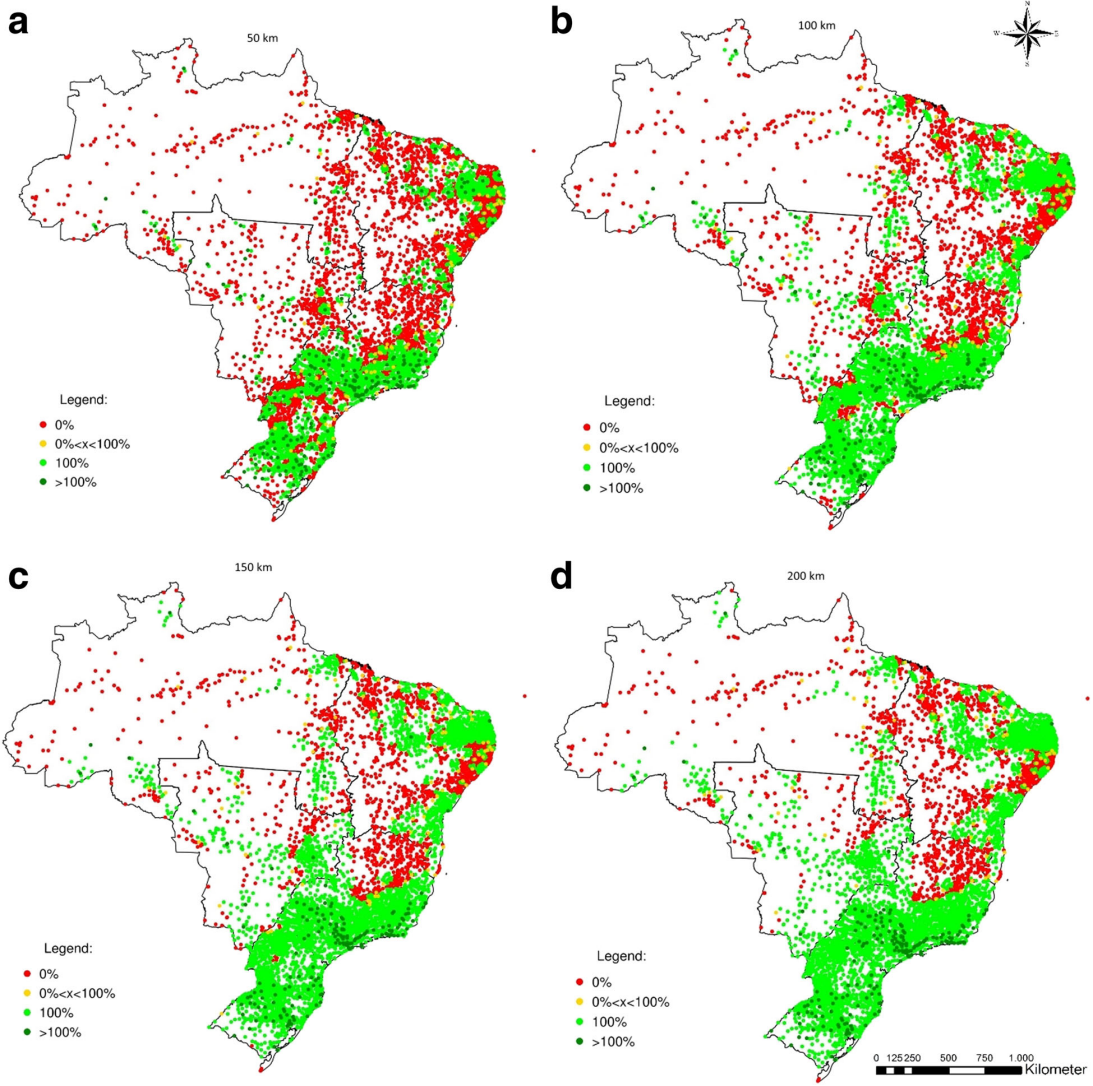
Percentage of the population served by the potential provision of bone densitometers

**Fig. 6:**
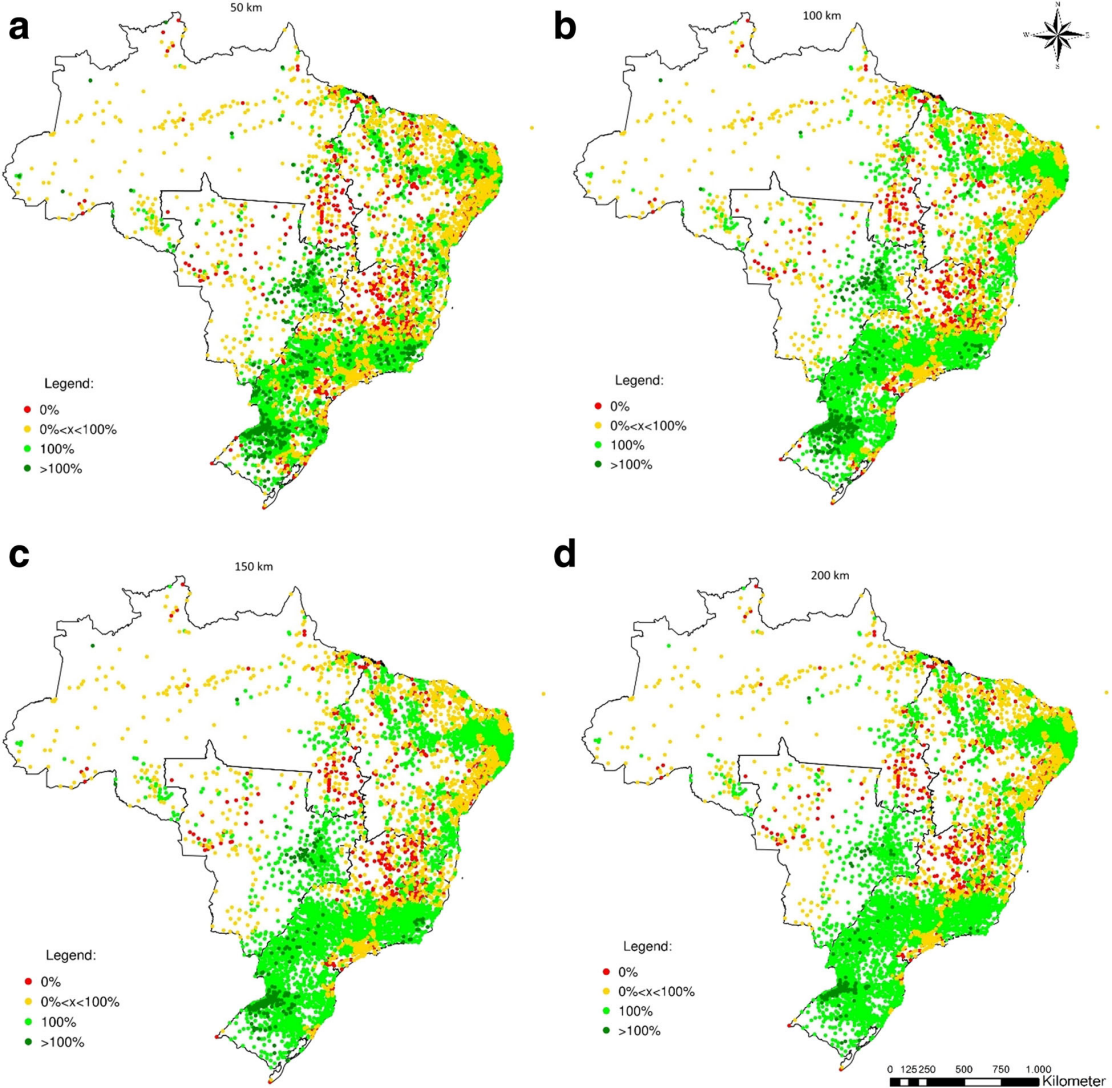
Percentage of the population served by the potential provision of hospital beds

**Fig. 7:**
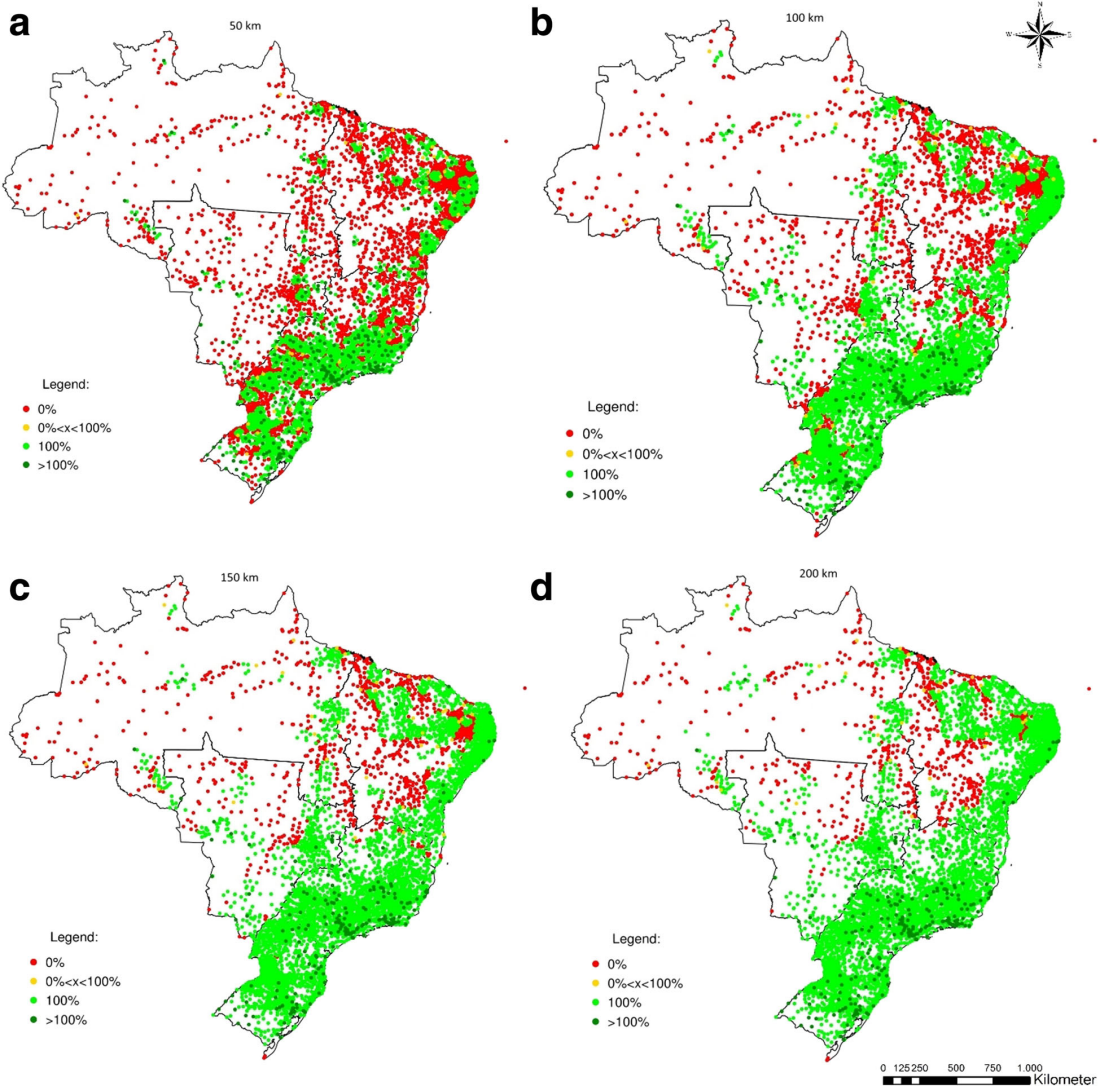
Percentage of the population served by the potential provision of dialysis machines

**Fig. 8:**
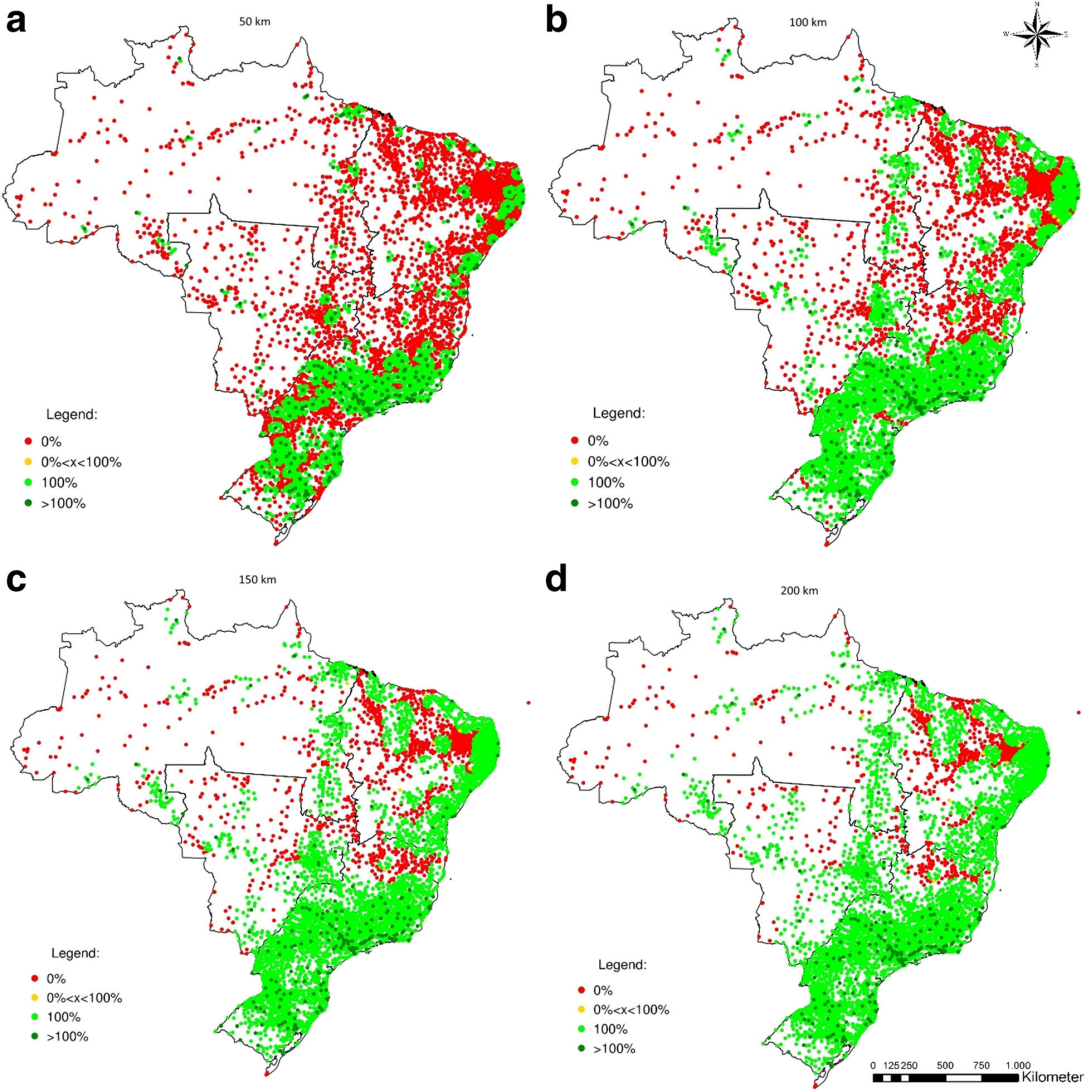
Percentage of the population served by the potential provision of MRIs

The figures shown above summarize results by state. However, when we actually look at the municipal level, both imbalances between and among states are highlighted. Figures 5, 6, 7 and 8 show the percentage of the population of each of the 5564 Brazilian municipalities that is covered by the provision of health equipment based on limits of 50 km (a), 100 km (b), 150 km (c) and 200 km (d) to their range of supply. Each circle on the map represents one municipality.

The spatial distribution of bone densitometers reveals a greater disparity between the Southern and Northern regions of Brazil (Fig. 5). Considering a maximum range of 50 km, 87.7% of the municipalities in the North, 77.7% in the Central-West, and 62.7% in the Northeast have 0% coverage, whereas in the Southeast and South these figures are 31.9% and 27.1%. If the maximum range is set to 200 km, the North and Northeast remain with 53.5% and 37.8% of municipalities with 0% coverage, respectively. The Central-West benefits from its proximity to the South and Southeast regions and has its 0% coverage ratio down to 19.7%. The South and Southeast present 0.2% and 13.3% rates of 0% coverage, respectively. Despite the lack of bone densitometers in the North, the South and Southeast regions contain 168 municipalities with excess supply under the range of 200 km (Table 2).

**Table 2:** Percentage of municipalities with no coverage for each equipment and maximum range by region (%)

Region	N	50 km	100 km	150 km	200 km
Municipalities		Bone densitometer			
North	450	87.8	68.4	57.5	53.5
Northeast	1794	62.7	46.7	40.9	37.8
Southeast	1668	32.0	20.6	15.4	13.3
South	1191	27.1	4.4	<1	<1
Centre-West	467	77.7	51.9	31.8	19.7
Brazil	5570	49.2	32.0	25.2	22.2
Hospital beds					
North	450	20.0	16.3	16.0	16.3
Northeast	1794	12.8	10.7	10.2	10.2
Southeast	1668	17.3	13.2	12.2	11.2
South	1191	5.0	2.6	1.4	<1
Centre-West	467	7.7	6.2	5.8	5.8
Brazil	5570	12.6	9.8	9.0	8.6
Dialysis machines					
North	450	82.6	59.9	52.8	50.3
Northeast	1794	63.9	38.6	27.4	18.1
Southeast	1668	27.8	5.4	1.4	<1
South	1191	28.2	6.0	<1	<1
Centre-West	467	74.9	44.8	27.7	17.6
Brazil	5570	47.9	23.9	15.9	11.5
MRIs					
North	450	81.5	55.0	38.1	23.4
Northeast	1794	77.0	48.4	32.3	21.0
Southeast	1668	33.5	13.5	7.4	4.8
South	1191	30.6	1.6	<1	<1
Centre-West	467	81.3	55.4	32.2	17.0
Brazil	5570	54.8	29.0	18.5	11.5

Regarding hospital beds (Fig. 6), several municipalities in the Central-West, Southeast, and South show an excess of supply hospital beds under a range of 50 km (>100% coverage). Concurrently, many others in the Northeast and North lack the provision of such equipment, and are either partially served (coverage between 0% and 100%) or completely not covered (coverage = 0%). Even if the maximum range is expanded to 200 km, it is possible observe municipalities in the North and in the Northeast not served. Nevertheless, as expected, the distribution of hospital beds in Brazil is the most spatially balanced of all equipment considered.

Figures 7 and 8 show the potential coverage of dialysis equipment and MRIs. Both types of equipment indicate a similar pattern. The provision of these types of equipment is highly concentrated when the maximum range is smaller, but both show comprehensive coverage when the range is greater, especially in South and Southeast. If the maximum range is 50 km, most of the North, Central-West and Northeast have 0% coverage of dialysis equipment - respectively 82.6%, 74.9%, and 63.9%, contrasted with 27.8% in the Southeast and 28.2% in the South. Even with a maximum range of 200 km, 50.3% of the municipalities in North, 18.1% in the Northeast, and 17.6% in the Central-West remain with 0% coverage. A total of 170 out of 212 municipalities (72.9%) with excess supply of dialysis machines are located in the South or Southeast with maximum range of 50 km. If 200Km is set as limit instead, this proportion is raised to 85.2% (Table 2).

With respect to MRIs, 45.3% of the municipalities with 0% coverage are in the Northeast with maximum range of 50 km. If it is increased to 200 km, the Northeast accounts for 58.6% of total municipalities with 0% coverage, while the Southeast accounts for only 12.5% and the South for 0.3%. It is worthy of mention that the Northeast contains 32% of all Brazilian municipalities, the Southeast 30%, and the South 21%.

## Discussion

This study evaluated the network of health equipment provision in Brazil and jointly analyzed the private and public sectors. We assess two main aspects of a spatially balanced health system: the regional availability of health equipment and the geographic distance between its demand and supply at a municipality level. The provision of hospital beds, bone densitometers, dialysis machine, and MRIs were selected as indicators.

Since most health equipment of intermediate and high complexities present economies of scale, their supply is expected to be spatially concentrated, constituting a network of provision of health care. In such a way, the centers of provision are expected to show an excess of supply which is enough to provide coverage for their influence areas. Due to the low level of mobility of the equipment, it is expected that the patients will travel from their original location to the locality where equipment is provided.

Our results show that the amount supplied for three of these types of equipment (namely bone densitometer, dialysis machines, and MRIs) exceeds the standard established by the Ministry of Health. Nevertheless, if limits are established to the range of provision, even wide ranges such as 200 km, several regions in Brazil lack the provision of the health equipment considered. Therefore, in the context of limited resources, the spatial distribution of items of equipment is an issue at least as important (if not more) as the amount of equipment provided.

The clusters of municipalities highlighted by the analysis contribute to support health policies to address unbalances in the distribution of health equipment. Despite the fact that MRI and dialysis equipment showed a similar pattern of clustering the impact of unbalances in the distribution of dialysis machine have more severe consequences for the access to health services. The use of dialysis machine by patients is performed nearly in a daily basis, demanding more effort to overcome large distances. Considering the 50 km distance, for example, many regions of the country face a lack of dialysis equipment capable to cover the population resident in municipalities of the North, Northeast and Central-West regions. The possibility to categorize the demand of a region accordingly to its equipment distribution can provide valuable data to policymakers elect priority areas for investment.

In our analysis, distance was the only factor that influences supply and demand. However, several other factors may affect access to the equipment. Although some places may be advantaged by an adequate provision of equipment, access to this equipment might be constrained by knowledge of their availability, costs, cultural barriers and transportation issues. In other words, availability of equipment alone is insufficient to guarantee access to it. Yet, availability is indeed a requirement for accessibility.

Furthermore we considered the provision of health equipment independent of whether the provider was from the public or private sectors. Consequently, our scope is restricted to the absolute provision of health equipment. The reason for this is twofold: data availability and individual’s preferences. Our main data source - DATASUS - provides information on the existence of equipment (which we use here), equipment currently in use, and equipment available for patients who receive care in SUS. However, the information on the latter has some problems. Equipment available for patients using SUS can be provided by both public and private institutions, according to use agreements. When the equipment is provided by private institutions to patients from SUS, the data does not take into account limits imposed on use, quotas, and what services are indeed available to patients from SUS. The actual access to these items of equipment is strongly related to the nature of the providers, among several other factors, and is outside of the scope of this study. Therefore, by including equipment jointly provided by the public and private sectors, we may overestimate the supply in areas where the private sector plays an important role.

The possibility of overestimating supply by no means invalidates our results. Such an overestimation is contingent on the private sector’s market share, but the regions identified with excess demand represent places where there is a relative absence of health equipment, whether provided by public or private institutions. These regions experiencing excess demand are the main concern of this study, since they are the ones with resource deprivation resulting regional imbalances in the provision of health services in Brazil.

Several papers consider a measure of `self-distance’, or internal distance, when estimating market potential [16, 17]. These estimations usually assume homogeneous distribution of their variable of interest and transportation cost throughout the area of the spatial units considered. Applied to Brazil, this assumption would be even more misleading than the disregard of the `self-distance’. The reason for this is the absence of data on urban areas for all Brazilian municipalities. The ratio between total area and urban area is uneven among municipalities in Brazil. Since there is no information on urban areas for all Brazilian municipalities, the disregard of the internal distance seems to be the less harmful choice.

The level of detail of our analysis allows us to confirm that the distribution of health equipment is inefficient not only between states, but also within states. Special attention is required in relation to the North and Central-West of Brazil. Given the very low population density in these regions, it is difficult to conceive a network of provision which is both socially fair and economically efficient. Different approaches to health care must be weighed. Regardless of the solution, what can be seen now is a lack of provision of health equipment even in the regions with higher population density. This applies specifically to the Northeast of Brazil, which has a population density similar to the South, but a very different situation for provision of health equipment.

## Conclusions

Our findings underscore that the provision of health equipment in Brazil is not balanced across its regions. Gaps and overlaps can be found in the network of provision of analyzed equipment, with some places presenting excess supply while others attempt to meet the needs of their population. Although we consider private and public service jointly, filling gaps in the network of provision of health services in Brazil is mainly an issue of public policy. As the Brazilian Unified Health System is based on the concept of guaranteed access to health for all citizens, with full coverage of medical needs and horizontal equity, a spatially balanced provision of equipment must be part of its agenda.

## Abbreviations

DATASUS: Information Technology Department of Brazilian Ministry of Health
IBGE: Instituto Brasileiro de Geografia e Estatística
MRI: Magnetic resonance imaging
SUS: United health system
